# Trafficking of Endogenous Immunoglobulins by Endothelial Cells at the Blood-Brain Barrier

**DOI:** 10.1038/srep25658

**Published:** 2016-05-06

**Authors:** Roberto Villaseñor, Laurence Ozmen, Nadia Messaddeq, Fiona Grüninger, Hansruedi Loetscher, Annika Keller, Christer Betsholtz, Per-Ola Freskgård, Ludovic Collin

**Affiliations:** 1 Roche Pharma Research and Early Development (pRED), Neurodegeneration and Regeneration, Roche Innovation Center Basel, Switzerland; 2 Institut de Génétique et de Biologie Moléculaire et Cellulaire (IGBMC), Institut Clinique de la Souris (ICS), Centre National de la Recherche Scientifique (CNRS)/Institut National de la Santé et de la Recherche Médicale INSERM/UdS, Collège de France, BP 10142, Strasbourg, France; 3 Division of Neurosurgery, University Hospital Zürich, Zürich University, Frauenklinikstrasse 10, CH-8091 Zürich, Switzerland; 4 Vascular Biology Program, Department of Immunology, Genetics and Pathology, Uppsala University, Uppsala, Sweden; 5 Department of Medical Biochemistry and Biophysics, Karolinska Institutet, Stockholm, Sweden

## Abstract

The Blood-Brain Barrier (BBB) restricts access of large molecules to the brain. The low endocytic activity of brain endothelial cells (BECs) is believed to limit delivery of immunoglobulins (IgG) to the brain parenchyma. Here, we report that endogenous mouse IgG are localized within intracellular vesicles at steady state in BECs *in vivo*. Using high-resolution quantitative microscopy, we found a fraction of endocytosed IgG in lysosomes. We observed that loss of pericytes (key components of the BBB) in *pdgf-b*^*ret/ret*^ mice affects the intracellular distribution of endogenous mouse IgG in BECs. In these mice, endogenous IgG was not detected within lysosomes but instead accumulate at the basement membrane and brain parenchyma. Such IgG accumulation could be due to reduced lysosomal clearance and increased sorting to the abluminal membrane of BECs. Our results suggest that, in addition to low uptake from circulation, IgG lysosomal degradation may be a downstream mechanism by which BECs further restrict IgG access to the brain.

Therapeutic antibodies hold considerable potential in both diagnosis and treatment of diseases^1^,^2^. However, their use for passive or active immunotherapy in the central nervous system (CNS) is limited by the blood–brain barrier (BBB). It is estimated that the BBB prevents over 95% of drugs, including large molecules such as immunoglobulins (IgG), from accessing the brain^3^. In mice, less than 0.1% of peripherally administered IgG reaches the brain parenchyma^4^. This function of the BBB is critical for maintenance of brain homeostasis and results from the unique properties of brain endothelial cells (BECs). These cells are distinguished from peripheral endothelial cells by the presence of particularly tight intercellular junctions that prevent paracellular transport, by the expression of specialized molecular transporters and receptors at the apical and basolateral membranes and by a higher pericyte coverage. Furthermore, they interact with CNS-specific cell types, such as astrocytes, microglia and neurons, which together form the functional neurovascular unit (NVU)^5^,^6^,^7^. The precise role of BECs in protecting the brain from peripheral protein influx has been extensively studied. However, intracellular sorting and transport through the transcytosis pathway in BECs remains largely unexplored^8^.

Morphological studies of the BBB using transmission electron microscopy (TEM) showed that exogenous horseradish peroxidase (HRP) was poorly internalized within BECs^9^. This observation led to the widely held view that a low rate of endocytosis is a hallmark of the BBB^3^,^5^,^6^. Specifically, it is believed that “minimal vesicular trafficking”^10^ may be responsible for minimizing the amount of IgG that reaches the brain parenchyma^11^. However, additional mechanisms downstream of uptake may be involved. Despite extensive research on the delivery of therapeutic antibodies to the brain, surprisingly little is known about transcytosis of IgG^4^,^12^,^13^,^14^. Most studies focusing on uptake and sorting of IgG have been performed in cultured cells and data showing that IgG is present within BECs *in situ* in the NVU is limited^15^.

In this study, we investigated the distribution of IgG at the BBB and in BECs. By using quantitative high-resolution confocal microscopy, we show for the first time that endogenous mouse IgG (mIgG), one of the main components of plasma^16^, is present in intracellular vesicles within BECs. At steady state, a fraction of mIgG is found in lysosomes. We observed that loss of pericytes in *pdgf-b*^*ret/ret*^ mice^17^ affects the intracellular distribution of endogenous mIgG and of a peripherally administered antibody in BECs. Our data suggest that pericytes modulate IgG trafficking by reducing their lysosomal transport in BECs. Overall, our results suggest that, in addition to a low basal rate of uptake, lysosomal degradation of IgG is a downstream mechanism by which BECs may limit the amount of IgG that enters the brain.

## Results

**Figure 1:**
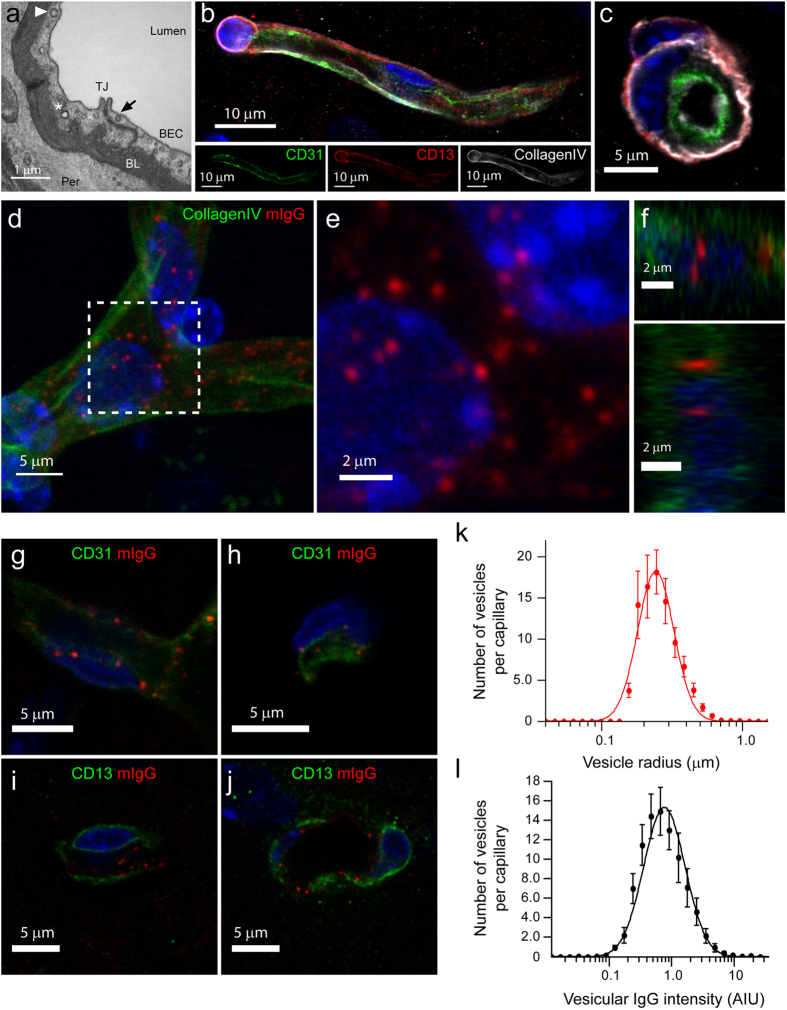
Intracellular localization of endogenous mIgG in brain endothelial cells. (**a**) Representative TEM cross-section of a brain cortical microvessel. The arrow points to a non-coated vesicle budding from the luminal membrane, the arrowhead points to a clathrin-coated-vesicle and the asterisk marks an intracellular vesicle. BEC, Brain Endothelial Cell; TJ, Tight Junction; BL, Basal Lamina; Per, Pericyte. (**b,c)** Representative 3D reconstruction of the NVU showing a BEC (marked by CD31 expression, green), Pericyte (marked by CD13 expression, red) within the Basal Lamina (CollagenIV, grey). The cross-section in the right panel (**c**) is a single optical section to highlight the vascular lumen. (**d–f**) Representative 3D reconstruction of a microvessel (marked by CollagenIV, green) with mIgG (red) in punctate structures (**d**). (**e**) shows a high magnification 3D reconstruction of the boxed area. Cross-sections in (**f**) show that vesicles are within the Basal Lamina and not in the parenchymal space. (**g–j**) Representative single optical sections of mIgG puncta localizing specifically within BECs marked with CD31 in green (**g,h**) but not in pericytes marked with CD13 in green (**i,j**). (**k,l**) Histograms for the radius of mIgG-positive intracellular vesicles (**k**) and normalized mIgG intensity per vesicle (**l**) in semi-logarithmic scale. Points show the mean value ± SEM of each size or intensity bin for 30 microvessels from 3 different C57BL/6 mice. The continuous line shows a log-normal fit of the experimental data. In all images, DAPI-stained nuclei are shown in blue.

We first applied a confocal light-microscopy protocol to image different cell types of the NVU. Our aim was to visualize intracellular structures that could thus far be detected only by electron microscopy (Fig. 1a). We reconstructed a 3D model of the NVU by immunofluorescent-labelling of BECs, pericytes and basal lamina markers (Fig. 1b,c; Table 1). Next, we examined the distribution of endogenous mIgG within the NVU. Under physiological conditions, it is believed that the low endocytosis rate of BECs is sufficient to exclude mIgG from the brain parenchyma^11^. Unexpectedly, we detected numerous mIgG puncta within capillaries (Fig. 1d–f; Supplementary Video 1). This distribution of mIgG was not an artefact caused by unspecific antibody binding since (i) we observed the same pattern using three different anti-mouse antibodies (Fig. 1d,g–j, Supplementary Fig. 1), (ii) no signal was observed using secondary antibodies against goat or human IgGs (Supplementary Figs 1 and 5), and (iii) the signal was restricted to the intracellular space in capillaries delineated by CollagenIV (Fig. 1d–f). We found that the distribution of mIgG was widespread along the vasculature in the cerebral cortex. However, the punctate pattern of mIgG was only evident at high-resolution (Supplementary Fig. 2). The majority of these puncta occurred within BECs and not pericytes, as shown by staining with CD31 (Fig. 1g,h) or CD13 (Fig. 1i,j).

The mIgG puncta had a radius between 0.2 and 0.4 μm with the majority below 0.5 μm, in agreement with the size of endosomes (Fig. 1k, Supplementary Fig. 3). We also estimated the amount of mIgG contained in individual puncta by quantifying the fluorescence intensity per puncta. Intensity values corresponding to individual puncta spanned nearly three orders of magnitude (Fig. 1l) suggesting that some puncta accumulate mIgG^18^.

**Figure 2:**
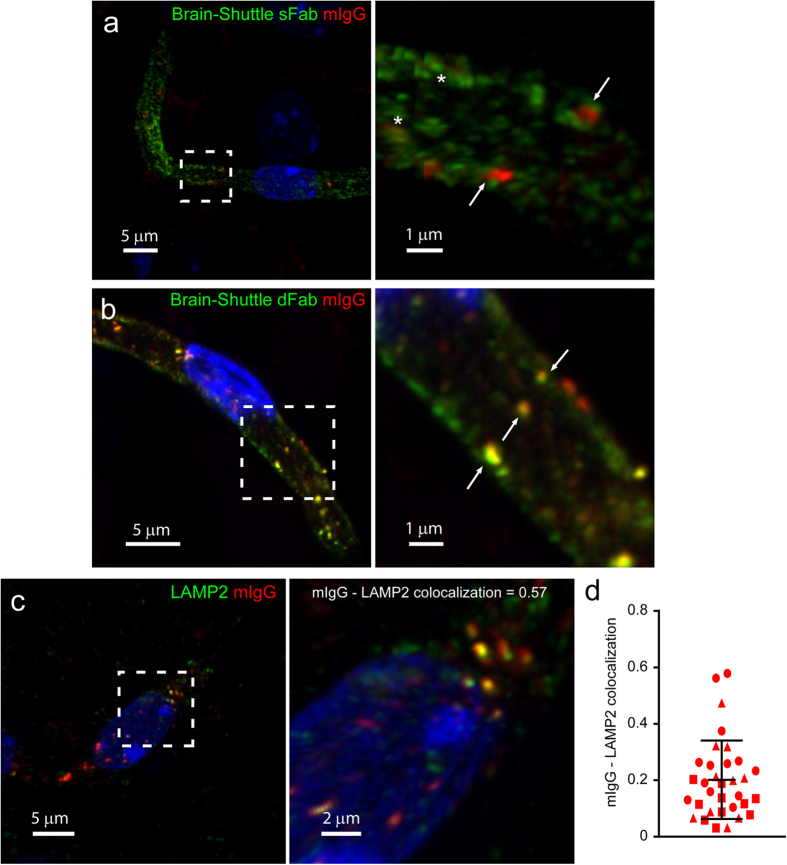
Endogenous mIgG localizes to lysosomes in BECs. (**a,b)** Representative 3D reconstruction of the intracellular distribution of the Brain Shuttle sFab (**a**) or dFab (**b**) (both in green) and endogenous mIgG (red) within a single brain microvessel. The right panels show high magnification image of the boxed area. Arrows highlight the minimal overlap between mIgG and BS-sFab vesicles in (**a**) and extensive colocalization between mIgG and BS-dFab in (**b**). Asterisks show Brain Shuttle-positive vesicles overlapping with faint mIgG vesicles. (**c**) Representative 3D reconstruction of the intracellular distribution of LAMP2-positive lysosomes (green) and endogenous mIgG (red) within a single brain microvessel. The right panel shows a high magnification image of the boxed area and highlights the colocalization between mIgG and LAMP2-positive lysosomes. In all images, DAPI-stained nuclei are shown in blue. (**d**) Colocalization of mIgG to LAMP2 expressed as the fraction of the total vesicular mIgG intensity colocalized with LAMP2 vesicles. Points show measurements from individual microvessels. Each symbol corresponds to a different animal. Lines show the mean ± SD for 33 microvessels from 3 different animals.

Next, we analyzed the localization of mIgG within BECs. Extensive antibody screening was performed to identify suitable endosomal markers compatible with our *ex vivo* technique. However, many antibodies gave non-specific punctate staining and were not considered for further analysis. As an alternative we exploited two recently described “Brain Shuttle” antibodies as markers of different endosomal populations^19^. Monovalent Brain Shuttle (BS-sFab) binds to the transferrin receptor (TfR) and is trafficked through the transferrin receptor-mediated transcytosis pathway, which includes both early and recycling endosomes, ultimately reaching the brain parenchymal space. Divalent Brain Shuttle (BS-dFab) also binds to the transferrin receptor but is trafficked for degradation in lysosomes^19^. The Brain Shuttles were detected using a secondary antibody against human IgG. Mice were administered with the different BS constructs (6 mg/kg) and sacrificed either 30 minutes (BS-sFab) or 8 hours (BS-dFab) post-injection. After 8 hours, BS-sFab is localized in the brain parenchyma with only minimal signal in capillaries and was thus not suitable for this analysis^19^. We found BS-sFab in vesicular structures within capillaries (Fig. 2a). Interestingly, we observed colocalization between BS-sFab and mIgG in vesicles close to the luminal membrane (Fig. 2a, asterisk). In contrast, there was little overlap between BS-sFab- and large mIgG-positive vesicles (Fig. 2a, arrows), suggesting that BS-sFab and mIgG are initially transported through a common endosomal compartment and that mIgG is subsequently sorted away from the canonical TfR transcytosis pathway. On the other hand, we observed extensive colocalization between BS-dFab and mIgG (Fig. 2b). Since BS-dFab is sorted to lysosomes this suggested that mIgG-positive vesicles are also transported to lysosomes^19^. To confirm this hypothesis, we performed a double immunostaining using an antibody against LAMP2, a lysosomal marker, and mIgG. We found that 20% of the total intracellular mIgG colocalized with lysosomes at steady state (Fig. 2c,d). In BECs, therefore, a substantial amount of mIgG is transported to lysosomes for degradation.

**Figure 3:**
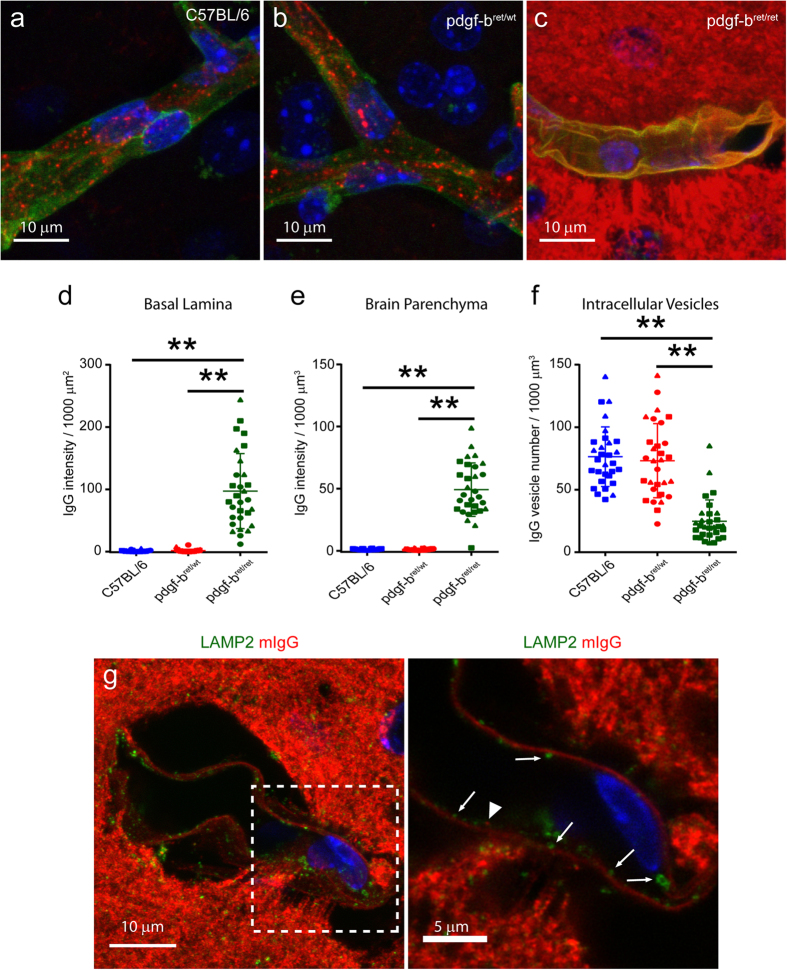
Relocalization of intracellular mIgG to the abluminal membrane in pericyte-deficient mice. (**a–c**) Representative maximum projection images of brain microvessels (marked by CollagenIV in green) showing the localization of mIgG (red) in C57BL/6 (**a**) *pdgf-b*^*ret/wt*^ (**b**) and *pdgf-b*^*ret/ret*^ (**c**) mice. DAPI-stained nuclei are shown in blue. (**d–f**) Graphs showing the quantification of mIgG intensity per μm^2^ of basal lamina (**d**) mIgG intensity per μm^3^ of brain parenchyma (**e**) and number of vesicles per μm^3^ of microvessel volume (**f**) in C57BL/6 (blue), *pdgf-b*^*ret/wt*^ (red), and *pdgf-b*^*ret/ret*^ mice (green). Points show measurements from individual microvessels. Each symbol corresponds to a different animal. Lines show the mean ± SD for 30 microvessels from 3 different animals for each phenotype. **p < 0.0001 by Fisher’s LSD test. (**g**) Representative maximum projection of a brain microvessel in *pdgf-b*^*ret/ret*^ mice showing the localization of mIgG (red) and lysosomes marked by LAMP2 (green). The right panel shows a high magnification of a single optical slice image of the boxed area and highlights the reduced colocalization between LAMP2 and mIgG. Individual mIgG and LAMP2 vesicles are marked with arrowheads and arrows, respectively.

Pericytes are key components of the NVU that are known to control the permeability of the BBB. Previous work showed that reduction of pericyte coverage of brain microcapillaries in *pdgf-b*^*ret/ret*^ mice results in the acute extravasation of injected tracers due to increased transcytosis^17^. However, the specific changes in the transcytosis pathway (for example uptake, sorting or fusion with the plasma membrane) were not explored. Therefore, we applied our imaging and quantification protocol to analyze the changes in endogenous mIgG transport in *pdgf-b*^*ret/ret*^ mice in comparison to control mice (Fig. 3a–c). We used CollagenIV, a marker of the basal lamina, to delineate capillaries in 3D and then quantified the amount of mIgG that accumulated within the basal lamina, the amount of mIgG that accumulated in the brain parenchymal space and the number of mIgG-positive vesicles contained within microcapillaries (Fig. 3d–f, respectively). The amount of endogenous mIgG increased 100-fold within the basal lamina and 50-fold in the brain parenchymal space of *pdgf-b*^*ret/ret*^ mice compared to both C57BL/6 and *pdgf-b*^*ret/wt*^ mice (Fig. 3a–e, Supplementary Fig. 4). The enlargement of capillaries in *pdgf-b*^*ret/ret*^ was previously reported (Fig. 3c)^17^. Strikingly, the number of mIgG-positive intracellular vesicular structures was significantly reduced in *pdgf-b*^*ret/ret*^ mice compared to C57BL/6 or *pdgf-b*^*ret/wt*^ mice (Fig. 3f). Moreover, the few remaining vesicular structures did not colocalize with LAMP2-positive lysosomes (Fig. 3g, inset and arrows). Therefore, the reduced number of intracellular mIgG-positive vesicles in *pdgf-b*^*ret/ret*^ mice could result from the increased transport and delivery of mIgG to the abluminal membrane.

**Figure 4:**
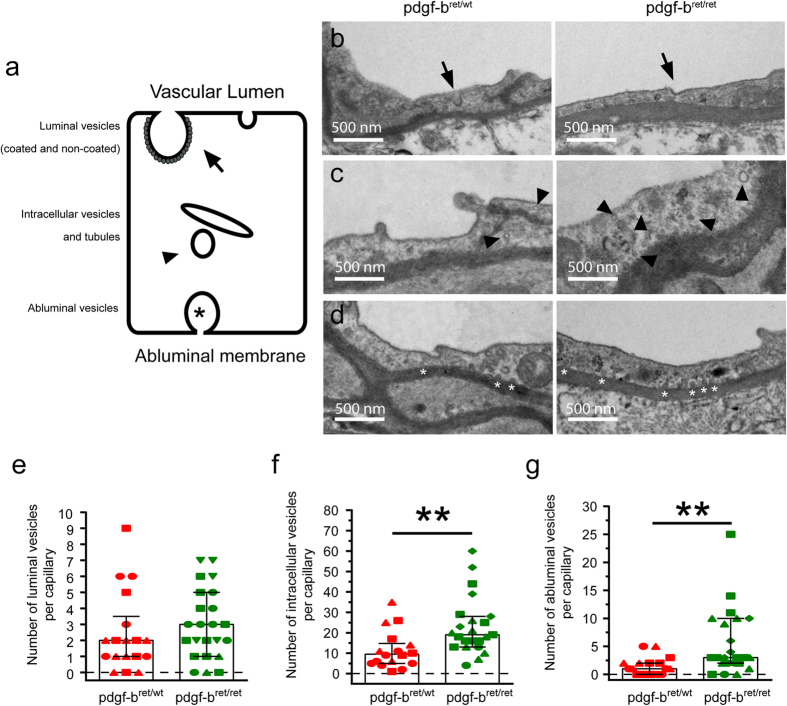
Increase of intracellular and abluminal vesicles in pericyte-deficient mice. (**a**) Scheme representing the different vesicle populations identified by TEM in BECs. (**b–d**) representative TEM cross-sections of brain microvessels in *pdgf-b*^*ret/wt*^ (left panel), and *pdgf-b*^*ret/ret*^ mice (right panel) showing coated and non-coated luminal vesicles (**b**) intracellular vesicles and tubules (**c**) and abluminal vesicles (**d**). (**e–g**) Graphs showing the quantification of the number of vesicles per capillary identified by TEM in *pdgf-b*^*ret/wt*^ (red), and *pdgf-b*^*ret/ret*^ mice (green). Points show measurements from individual microvessels. Each symbol corresponds to a different animal. Columns represent the median and error bars the interquartile range for at least 18 microvessels from at least 3 different animals per phenotype. **p < 0.005 by Mann-Whitney U test.

To test this hypothesis, we used TEM to analyze the distribution of the total pool of intracellular vesicles in *pdgf-b*^*ret/ret*^ mice (Fig. 4a). Vesicles were classified as (i) luminal, including coated and non-coated vesicles docked or budding from the luminal membrane (Fig. 4b), (ii) intracellular, including tubules and vesicles within the cytoplasm (Fig. 4c) or (iii) abluminal i.e. vesicles docked to the abluminal membrane (Fig. 4d). There was no significant difference between luminal vesicles in *pdgf-b*^*ret/ret*^ and *pdgf-b*^*ret/wt*^ mice (Fig. 4e). However, both the number of intracellular and abluminal vesicles were significantly increased in *pdgf-b*^*ret/ret*^ BECs (Fig. 4f,g). These observations suggest that the increased delivery of endogenous mIgG to the brain parenchyma upon pericyte depletion results from an increased intracellular transport to the abluminal membrane.

**Figure 5:**
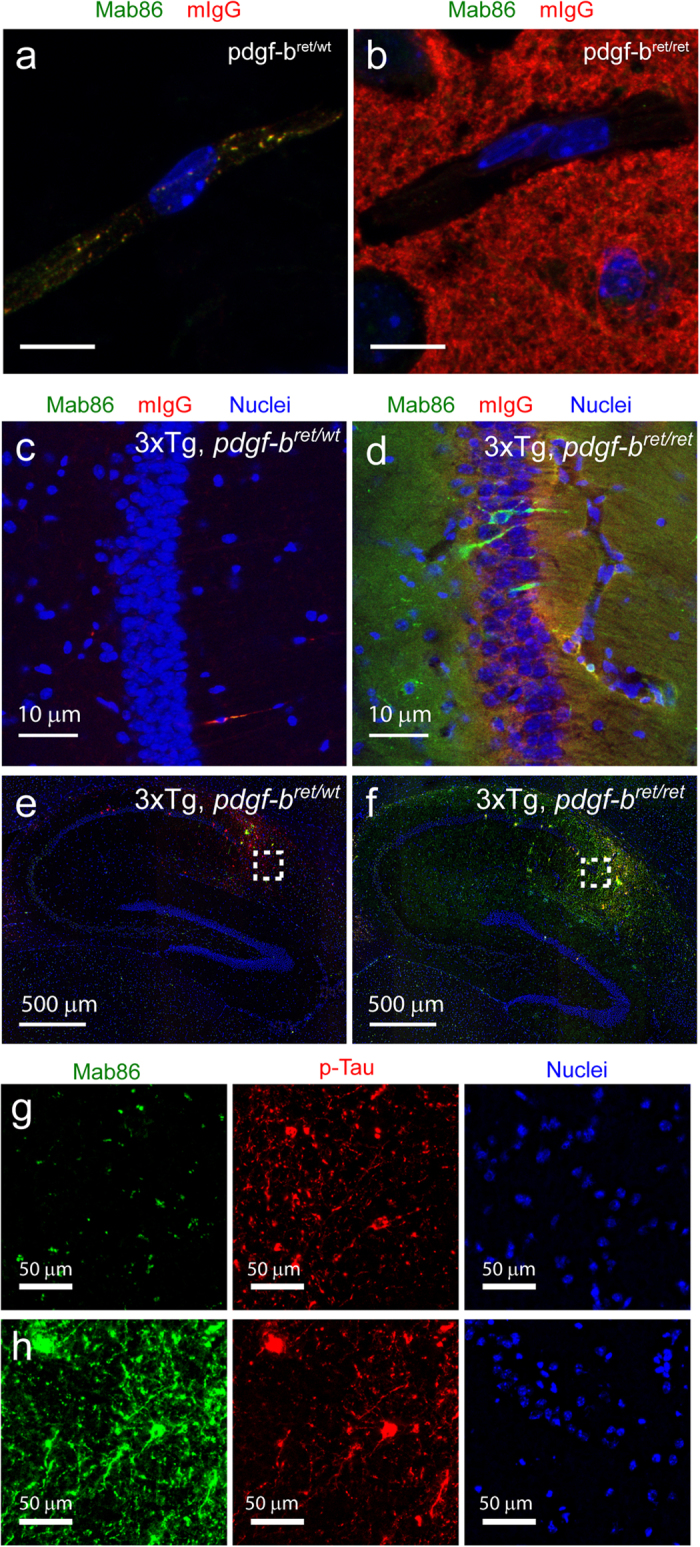
Pericyte depletion results in increased target engagement of an antibody against phosphotau. (**a,b)** Representative maximum intensity projections of microcapillaries comparing the intracellular distribution of Mab86 (green) after acute injection and endogenous mIgG (red) in *pdgf-b*^*ret/wt*^ (**a**), and *pdgf-b*^*ret/ret*^ (**b**) mice. (**c,d**) High-resolution images of neurons on the hippocampus of 3Tg x *pdgf-b*^*ret/wt*^ (**c**) or 3Tg x *pdgf-b*^*ret/ret*^ (**d**) mice. In all images, DAPI-stained nuclei are shown in blue. (**e,f**) Representative low-magnification images of the hippocampus of 3Tg x *pdgf-b*^*ret/wt*^ (**e**) or 3Tg x *pdgf-b*^*ret/ret*^ (**f**) mice showing phosphotau positive neurons (red) and Mab86 (green). (**g,h**) High magnification images of the boxed area in 3Tg x *pdgf-b*^*ret/wt*^ (**g**) or 3Tg x *pdgf-b*^*ret/ret*^ (**h**) mice highlighting the accumulation of Mab86 in phosphotau positive neurons in 3Tg x *pdgf-b*^*ret/ret*^ mice.

To further confirm these data, we investigated the intracellular distribution in BECs of a human IgG following peripheral administration to *pdgf-b*^*ret/ret*^ and *pdgf-b*^*ret/wt*^ mice. Mab86 is a humanized antibody that binds specifically to phosphorylated tau^20^. One hour post-injection, Mab86 was localized within vesicular structures in *pdgf-b*^*ret/wt*^ BECs where it colocalized with endogenous mIgG (Fig. 5a, Supplementary Fig. 5). On the contrary, we did not detect Mab86-containing vesicular structures in pdgf*-b*^*ret/ret*^ BECs (Fig. 5b).

Finally, we generated a new mouse model by crossing *pdgf-b*^*ret/ret*^ animals with the TauPS2APP triple transgenic mouse model (3Tg) of Alzheimer’s Disease (AD)^21^ to test whether pericyte loss would increase transcytosis and delivery of Mab86 to phosphotau-containing hippocampal neurons. We injected 13 months-old mice i.p. with 30 mg/kg of Mab86 and analyzed brain sections 48 hours post-injection. Neither Mab86 nor mIgG were detectable in the brain parenchyma of 3Tg x *pdgf-b*^*ret/wt*^ mice (Fig. 5c). In 3Tg x *pdgf-b*^*ret/ret*^ mice, Mab86 and mIgG diffusely localized in the parenchymal space (Fig. 5d). However, Mab86 specifically accumulated in hippocampal neurons (Fig. 5d). We then examined tau pathology in the hippocampus of 3Tg x *pdgf-b*^*ret/wt*^ and 3Tg x *pdgf-b*^*ret/ret*^ mice and found that phosphotau localized predominantly at the tip of the CA1 region and the subiculum as previously described (Fig. 5e,f)^20^,^21^. In 3Tg.x *pdgf-b*^*ret/wt*^mice, very few Mab86-positive cells were detected (Fig. 5e,g). However, Mab86 target engagement was strongly enhanced in phosphotau positive neurons of 3Tg x *pdgf-b*^*ret/ret*^ mice (Fig. 5f–h). These data confirm that pericyte depletion increases the delivery of a functional IgG to the brain. Overall, our results support the hypothesis that increased IgG transcytosis to the brain in *pdgf-b*^*ret/ret*^ mice results from an increased intracellular trafficking to the abluminal membrane.

## Discussion

Earlier studies using electron microscopy showed that vesicular transport is extremely low in BECs, providing one possible explanation for how the BBB limits entry of peripheral proteins to the brain^3^,^5^,^9^. However, although TEM allows detailed characterization of the morphological properties of BECs and the NVU, it has limitations for the study of transcytosis. In particular, the number of intracellular vesicles within endothelial cells observed by TEM gives no information on the rates of endosome transport. A recent review on TEM studies found only a weak correlation between intracellular vesicles and transport capacity in different types of vascular bed^22^. This consideration prompted us to further investigate the mechanisms that restrict protein transport into the brain parenchyma.

We used TEM to quantify the total number of vesicular structures contained within BECs in *pdgf-b*^*ret/ret*^ and *pdgf-b*^*ret/wt*^mice. Whereas luminal vesicle numbers were similar in *pdgf-b*^*ret/ret*^ and *pdgf-b*^*ret/wt*^mice, pericyte loss in *pdgf-b*^*ret/ret*^ mice leads to increased numbers of intracellular vesicles. Docking and fusion of these vesicles with the abluminal membrane probably contributes to the increased protein permeability of the BBB as previously described^17^. To complement this analysis, we tried to identify the specific location of endogenous IgG at the NVU using quantitative high-resolution confocal imaging. In control mice, BECs contained numerous intracellular vesicles filled with varying amounts of mIgG. The steady state vesicular distribution observed *ex vivo* reflects the balance of uptake, endosome fusion, recycling, and degradation that has previously been described in *in vitro* models for cargo(s) such as LDL^18^. Importantly, our observations do not challenge the fact that BECs have a low basal uptake from circulation compared with peripheral endothelial cells. However, despite such a low uptake there are abundant mIgG-positive vesicles in BECs. Therefore, mechanisms downstream of uptake must exist to prevent the delivery of this intracellular mIgG pool to the brain parenchyma. First, mIgG may interact with FcRn and get recycled to the plasma membrane and released in the blood stream. FcRn is the only Fc receptor known to be expressed by BECs^23^. *In vitro,* FcRn-expressing endothelial cells can recycle IgG via Rab11-positive endosomes^15^. Therefore, binding of mIgG to FcRn within BEC endosomes could prevent their sorting to the abluminal membrane. Additional experiments using specific markers of FcRn recycling pathway would be required to confirm this hypothesis. Second, mIgG may be prevented from reaching the parenchyma through trafficking to lysosomes for degradation. In a series of *in vivo* experiments, Broadwell *et al* showed that circulatory proteins are endocytosed and eventually degraded in lysosomes^24^. Interestingly, we observed significant colocalization of mIgG with both LAMP2 (a lysosomal marker) and BS-dFab (gets trafficked to lysosomes) indicating that lysosomal degradation prevents delivery of mIgG to the brain. Together, these data suggest that lysosomal clearance of proteins in BECs may limit protein access to the brain parenchyma. Additional work using quantitative analysis of transport kinetics across multiple endosomal compartments *in vivo* will be required to fully characterize the sorting pathway for IgG across the BBB.

Previous work showed that pericytes are key regulators of BBB permeability^17^,^25^. For example, the transcytosis flux is increased in pericyte-deficient mice such as *pdgf-b*^*ret/ret*^^17^. We observed a significant reduction in the number of intracellular mIgG-positive vesicles in *pdgf-b*^*ret/ret*^ mice compared to control mice. Conversely, mIgG delivery to the brain was significantly increased. This could be due to an unchanged, saturated IgG uptake together with an increased release of mIgG to the brain in *pdgf-b*^*ret/ret*^ BECs^13^. Supporting this hypothesis we found (i) no difference in the number of luminal coated and non-coated vesicles and (ii) a significant increase in the number of abluminal vesicles in *pdgf-b*^*ret/ret*^ mice compared to control mice. These data suggest that pericyte loss may lead to increased vesicular flux to the abluminal membrane of BECs. The findings do not exclude that other fluid-phase uptake pathways^26^ are altered upon pericyte depletion^25^,^27^ but suggest that pericytes act on specific endocytosis pathways.

The BBB poses a formidable obstacle for delivery of therapeutic antibodies since less than 0.1% of peripherally administered IgG reaches the brain parenchyma^4^. We show for the first time that a systemically administered antibody, Mab86, can be detected in vesicular structures within BECs. The low brain exposure of Mab86 and its colocalization with endogenous mIgG in BECs suggests that lysosomal clearance may limit Mab86 transport to the brain parenchyma. Loss of pericytes significantly enhanced Mab86 brain delivery as shown by substantially increased target engagement in neurons and, similar to endogenous mIgG, did not increase the number of intracellular Mab86-positive vesicles in BECs. Overall our results suggest that pericytes regulate IgG transcytosis in part by controlling trafficking to lysosomes for degradation. Our data suggest that strategies to reduce IgG lysosomal clearance in BECs may enhance delivery of antibodies across the BBB and into the brain.

## Methods

### Mice

TauPS2APP (3Tg) and *pdgf-b*^*ret/ret*^ mice were described previously^17^,^21^. 3Tg mice were crossed with *pdgf-b*^*ret/wt*^mice to generate 3Tgx *pdgf-b*^*ret/ret*^ and 3Tg x *pdgf-b*^*ret/wt*^ littermates. All animal experiments were approved by the Swiss Veterinary Office Basel-Stadt and were carried out in accordance with the approved guidelines described in the Swiss animal permission #1902.

### Brain sectioning and immunofluorescence

Brain processing was performed as previously described^19^. Briefly, 19–20 months-old C57BL/6, *pdgf-b*^*ret/wt*^ or *pdgf-b*^*ret/ret*^ mice were euthanized with CO_2_ and transcardially perfused with PBS at 37 °C, followed by perfusion with 2% PFA. The brain was then removed and incubated overnight in 2% PFA at 4 °C before sectioning. Brains were included in 2% agarose and 100 μm sagittal sections were cut using a Leica VT1000M vibratome. Sections were stored at −20 °C in 1:1 PBS/Glycerol. Sections were processed for immunofluorescence by washing with PBS and permeabilization with PBS 0.3% Triton X-100 and 10% donkey serum for blocking. Primary antibodies were diluted in 5% donkey serum in PBS and incubated with sections for 72 hours at 4 °C, followed by washing with PBS and 1 hour incubation at room temperature with appropriate fluorescently-labelled secondary antibodies (Donkey anti-goat, donkey anti-rabbit, or donkey anti-rat IgG coupled to AlexaFluor488, 555, or 647, from LifeTechnologies) in 5% PBS. Finally, sections were washed with PBS, stained with 1 μg/ml DAPI and mounted using DAKO Fluorescent Mounting medium on glass slides with a 0.17 mm coverslip. Table 1 shows the list of antibodies used for the study.

### Electron microscopy

Brain samples were fixed by immersion in 2.5% glutaraldehyde and 2.5% paraformaldehyde in cacodylate buffer (0.1 M, pH 7.4) and washed in cacodylate buffer for 30 minutes. The samples were post-fixed in 1% osmium tetroxide in 0.1 M cacodylate buffer for 1 hour at 4 °C and dehydrated through graded alcohol (50, 70, 90, and 100%) and propylene oxide for 30 minutes each. Next, samples were oriented longitudinally and embedded in Epon 812. Ultrathin 70 nm sections were contrasted with uranyl acetate and lead citrate and examined at 70 kv with a Morgagni 268D electron microscope. Digital images were acquired with a Mega View III camera (Soft Imaging System). Cortical capillaries were selected randomly for quantification of intracellular vesicles.

### Imaging of intracellular mIgG at the NVU

Mounted brain sections were imaged using the Leica TCS SP8 microscope using a HC PL APO 63x/1.4 oil objective. Images were 12-bit and either 1024 × 1024 with 90 nm pixel size or 512 × 512 with 46.46 pixel size. Laser intensity and detector gain settings were optimized to minimize pixel saturation and maximize dynamic range. Between 15 and 20 optical sections were acquired per vessel covering a z-distance of 7–12.5 μm. Deconvolution of confocal images was performed using the Leica LAS-AF 3D deconvolution tool. Movies and 3D reconstructions of vessels were performed using Imaris. Cross-sections were performed using FIJI.

### Quantification of intracellular mIgG

Two different methods were used to quantify intracellular mIgG within BECs. Since the density of mIgG-positive structures was low and the z-stack acquired was only 7 μm thick, we could analyze the maximum intensity projection of each microvessel. Quantitative multiparametric image analysis (QMPIA) was performed using Kalaimoscope MotionTracking as previously described^18^,^28^,^29^. Object-based colocalization was estimated after substraction of random colocalization as previously described^30^. To quantify and compare the accumulation of mIgG in intracellular vesicles, basal lamina and parenchymal space, quantification were performed using Imaris. 3D-reconstructed images were segmented using an absolute intensity threshold mask on CollagenIV to identify intracellular structures (within CollagenIV mask), Basal Lamina (colocalized with CollagenIV mask) and parenchymal signal (outside of CollagenIV mask). Intracellular vesicles were segmented using Imaris spot detection algorithm with default parameters and an estimated diameter of 0.8 μm. mIgG intensity values were normalized by the surface area of the mask or the parenchymal volume. The total number of vesicles was normalized by the volume of CollagenIV mask. Quantifications were obtained from 10 microvessels per animal using at least 3 animals per genotype. Multiple statistical comparisons were performed using Fisher’s LSD test in GraphPad.

### Detection of BS or Mab86

14-months old TAuPS2APP mice were injected with 6 mg/kg BS i.v. and euthanized by pentobarbital 30 minutes after injection. Then, animals were transcardially perfused with PBS, followed by perfusion with 2% PFA. The brains were removed and incubated overnight in 2% PFA at 4 °C before sectioning. For Mab86 detection, 13 months old 3Tg x *pdgf-b*^*ret/wt*^ or 3Tg x *pdgf-b*^*ret/ret*^ mice were injected with 30 mg/kg Mab86 and euthanized by pentobarbital 1 or 48 hours after injection. Whole brain images were acquired from frozen sections prepared and immunostained with an anti-human PHF-TAU AT8 antibody and a goat anti-human IgG (H + L) AlexaFluor555 (LifeTechnologies) to detect Mab86 (Pierce, MN1020) as previously described^20^. Imaging was performed using a Metafer 4 slide scanning system (MetaSystems).

## Additional Information

**How to cite this article**: Villaseñor, R. *et al.* Trafficking of Endogenous Immunoglobulins by Endothelial Cells at the Blood-Brain Barrier. *Sci. Rep.* **6**, 25658; doi: 10.1038/srep25658 (2016).

## Supplementary Material

Supplementary Information

Supplementary Video 1

## Figures and Tables

**Table 1:** List of validated antibodies for immunofluorescence staining of free-floating sections.

Cell Type or Antigen	Antibody manufacturer
BECs (CD31/PECAM)	Rat monoclonal MEC13.3, Novus Biologicals
Pericytes (CD13)	Goat polyclonal, R&D Systems
Basal Lamina (CollagenIV)	Rabbit polyclonal, Serotec
Lysosomes (LAMP2)	Rat monoclonal ABL93, Fitzgerald
mIgG	Donkey anti-mouse IgG (H + L) AlexaFluor594, LifeTechnologies. Donkey anti-mouse IgG (H + L) AlexaFluor488, LifeTechnologies. Goat anti-mouse IgG (H + L) AlexaFluor555, LifeTechnologies
Brain Shuttle (huIgG)	Goat anti-human IgG (H + L) AlexaFluor555, LifeTechnologies. Goat anti-human IgG (H + L) AlexaFluor488, LifeTechnologies
