# Supplementary material for: Trafficking of Endogenous Immunoglobulins by Endothelial Cells at the Blood-Brain Barrier
Source: Sci Rep. 2016 May 6;6:25658. doi: 10.1038/srep25658 (PMC4858719; doi:10.1038/srep25658)
Supplement: Supplementary Information [file srep25658-s1.pdf]

Title: Trafficking of Endogenous Immunoglobulins by Endothelial Cells at the Blood-Brain Barrier

Roberto Villaseñor1, Laurence Ozmen1, Nadia Messaddeq2, Fiona Grüninger1, Hansruedi Loetscher1 Annika Keller3, Christer Betsholtz4, Per-Ola Freskgård1, Ludovic Collin1*

1. Roche Pharma Research and Early Development (pRED), Neurodegeneration and Regeneration, Roche Innovation Center Basel, Switzerland.
2. Institut de Génétique et de Biologie Moléculaire et Cellulaire (IGBMC), Institut Clinique de la Souris (ICS), Centre National de la Recherche Scientifique (CNRS)/Institut National de la Santé et de la Recherche Médicale INSERM/UdS, Collège de France, BP 10142, Strasbourg, France.
3. Division of Neurosurgery, University Hospital Zürich, Frauenklinikstrasse 10, CH-8091 Zürich, Switzerland.
4. Vascular Biology Program, Department of Immunology, Genetics and Pathology, Uppsala University, Uppsala, Sweden, and the Department of Medical Biochemistry and Biophysics, Karolinska Institutet, Stockholm, Sweden.

Supplementary Information

Supplementary video 1. Specific detection of mIgG only with anti-mouse IgG antibodies. Representative 3D reconstruction of a microvessel (marked by CollagenIV, green) with mIgG (red) in intracellular vesicles. Nuclei are shown in blue.

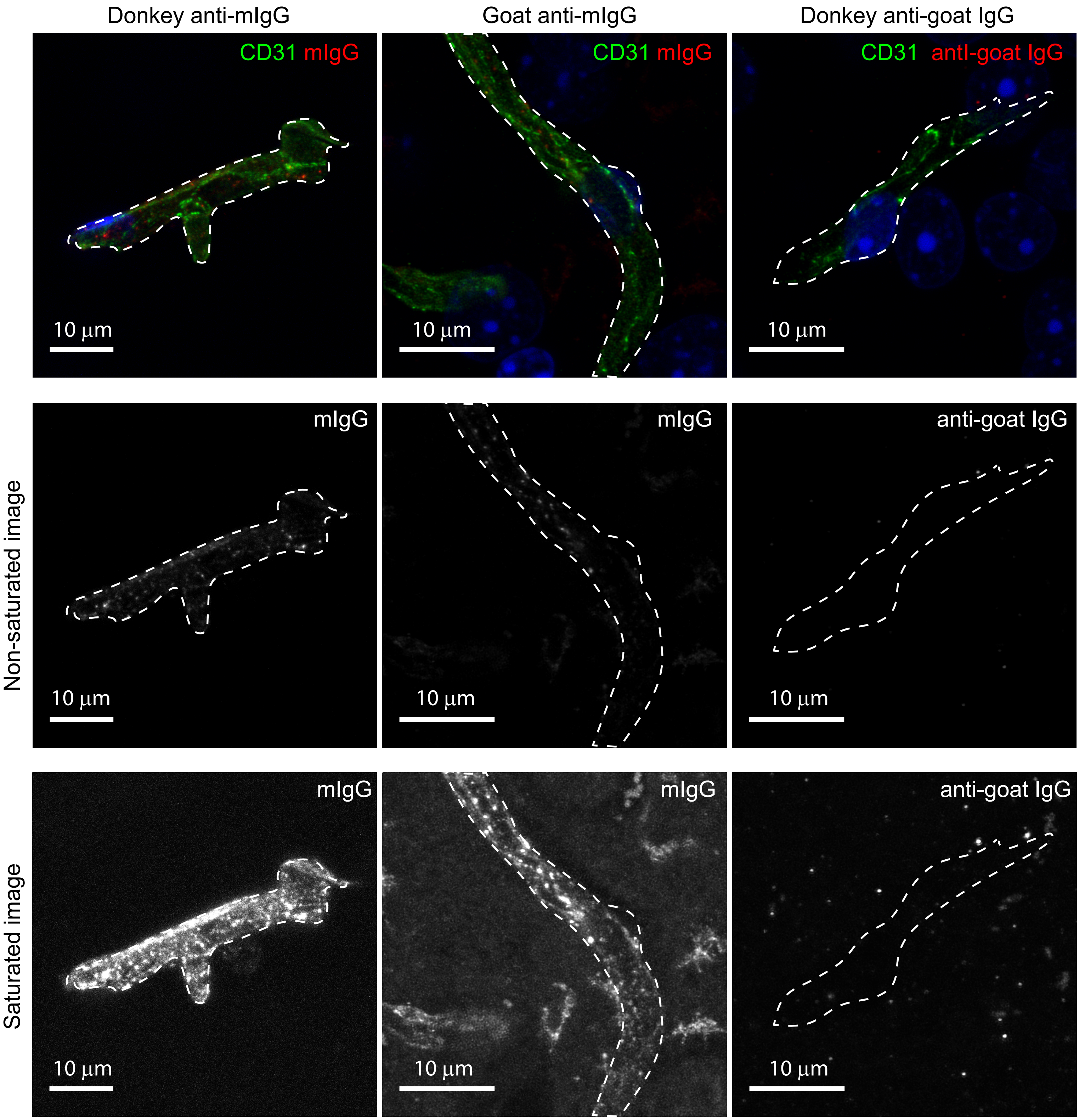

Supplementary Figure 1. Specific detection of mIgG using anti-mouse IgG antibodies. Representative high-resolution images of a cortical mouse microvessel showing that vesicular structures (red) are only detected after immunostaining with a donkey or goat anti-mouse IgG antibody (left and central panels) but not with a donkey anti-goat IgG antibody (right panel). Capillaries are identified using the endothelial marker CD31 in green. mIgG is shown in red and DAPI-stained nuclei in blue. The middle panels show non-saturated greyscale images. The bottom panels show saturated greyscale images to highlight the background staining pattern of anti-goat IgG.

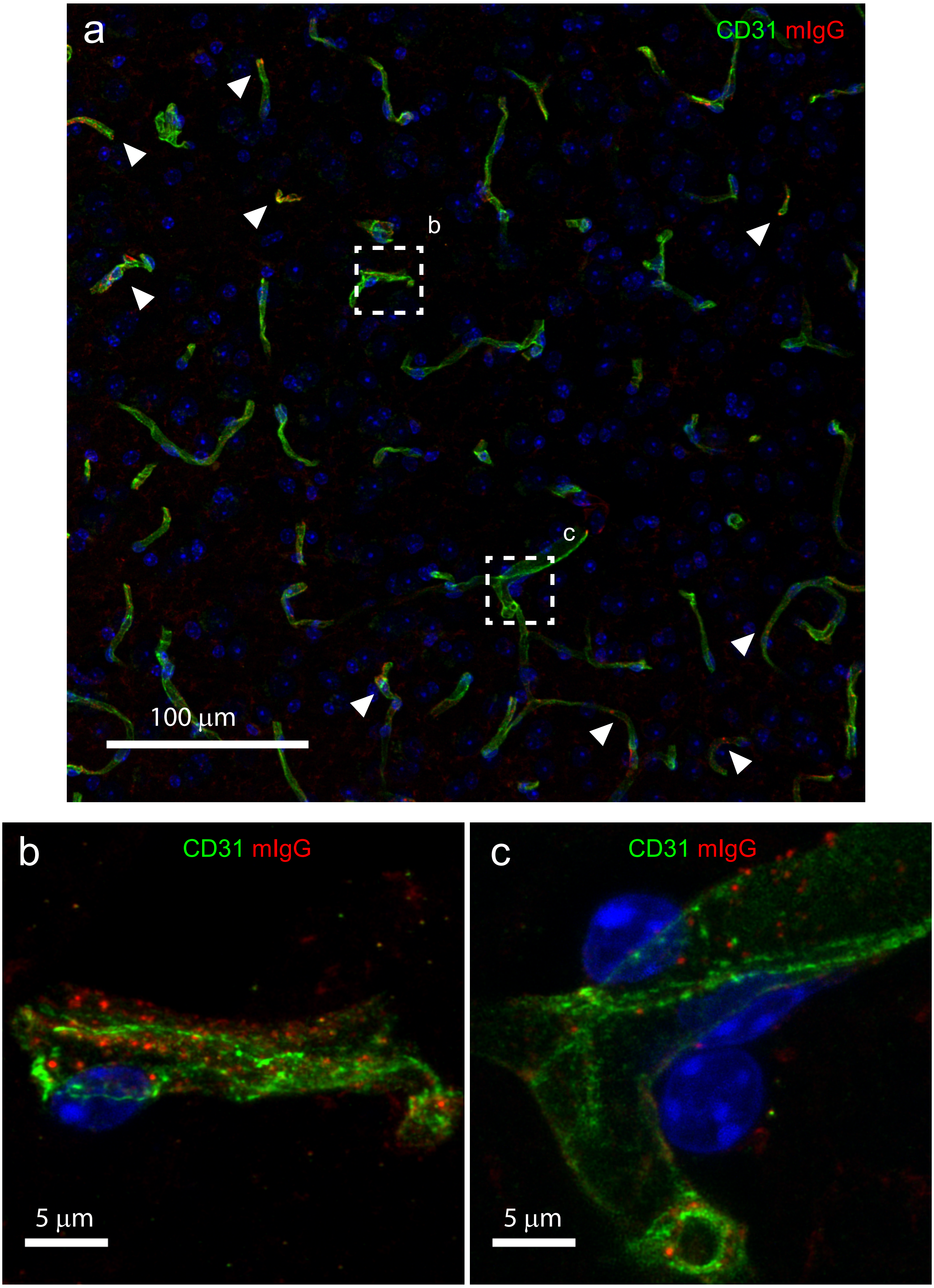

Supplementary Figure 2. Widespread distribution of mIgG-positive vesicles in brain vascular network. a, Representative low resolution image of a cortical region showing microcapillaries identified by CD31 in green and mIgG in red. The majority of capillaries show mIgG signal. Arrowheads indicate strong mIgG signal. **b,c**, High magnification of the boxed areas to show the distribution of mIgG puncta within capillaries.

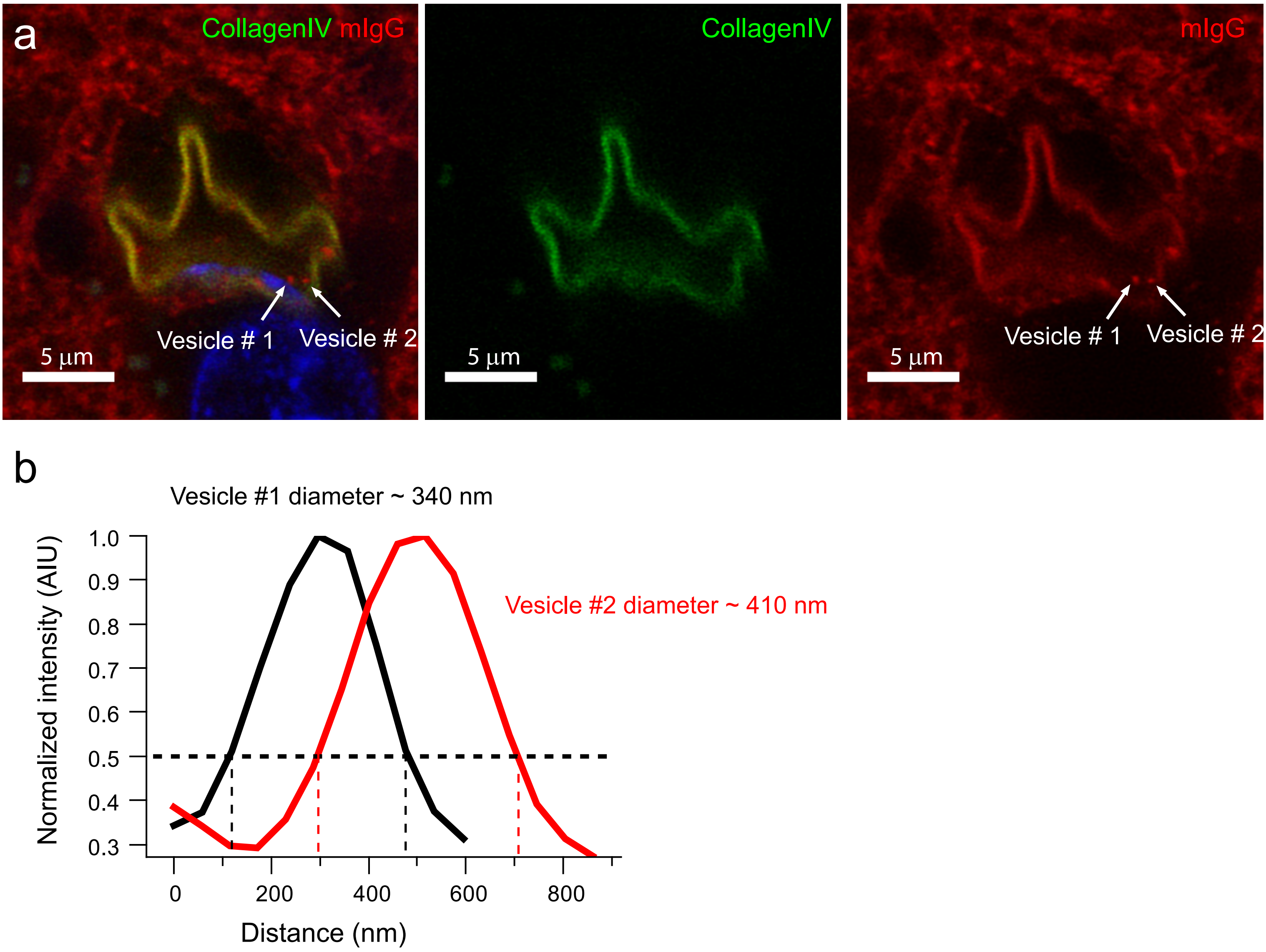

Supplementary Figure 3. Detection of small vesicles within microcapillaries of *pdgf-b*$^{ret/ret}$ mice. **a**, Representative single optical cross section of a microcapillary in pdgf-bret/ret mice with mIgG shown in red and CollagenIV in green. Arrows point to vesicles within the microcapillary. **b**, Line scan intensity profile of the two vesicles in **a**. The diameter was calculated from the Full-width half-maximum of both curves.

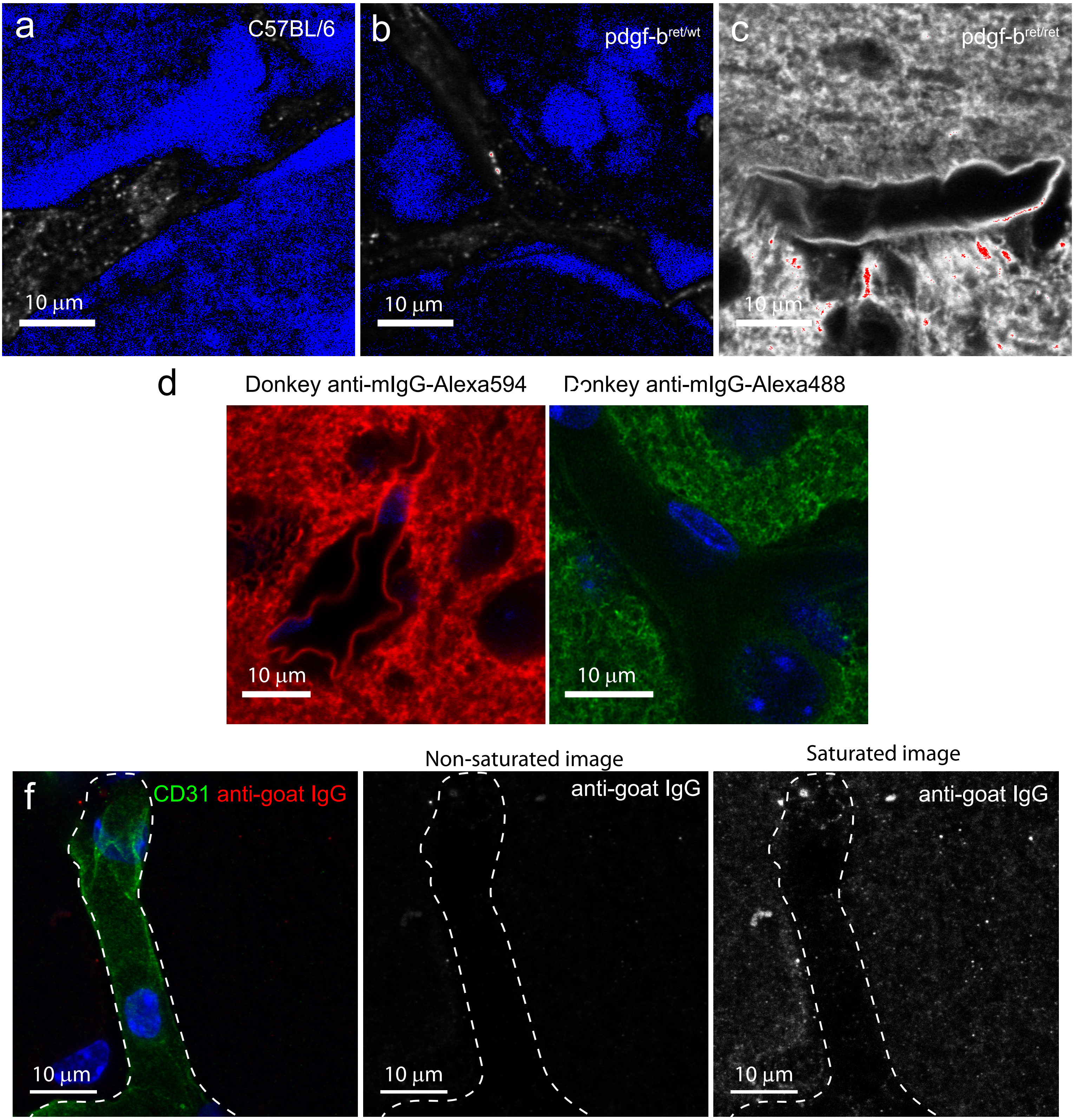

Supplementary Figure 4. Detection of non-saturated mIgG signal in *pdgf-b$^{ret/ret}$* mice. a-c, Single optical sections corresponding to the same maximum projection images shown in Figure 3a-c. Images are shown using ImageJ HiLo lookup table where saturated pixels are shown in red and empty pixels in blue. d-e, Single optical section of microcapillaries in pdgf-bret/ret mice using donkey anti-mIgG-Alexa594 (**d**) or donkey anti-mIgG-Alexa488 (**e**) showing that the same pattern of parenchymal IgG accumulation is detected with different fluorescently labelled antibodies. **f**, Representative maximum intensity projection of a microcapillary (marked by CD31, green) in pdgf-bret/ret mice showing that only background parenchymal signal is observed after immunofluorescent staining with a donkey anti-goat IgG antibody. The middle panel shows non-saturated greyscale images. The right shows saturated greyscale images to highlight the background staining pattern of anti-goat IgG.

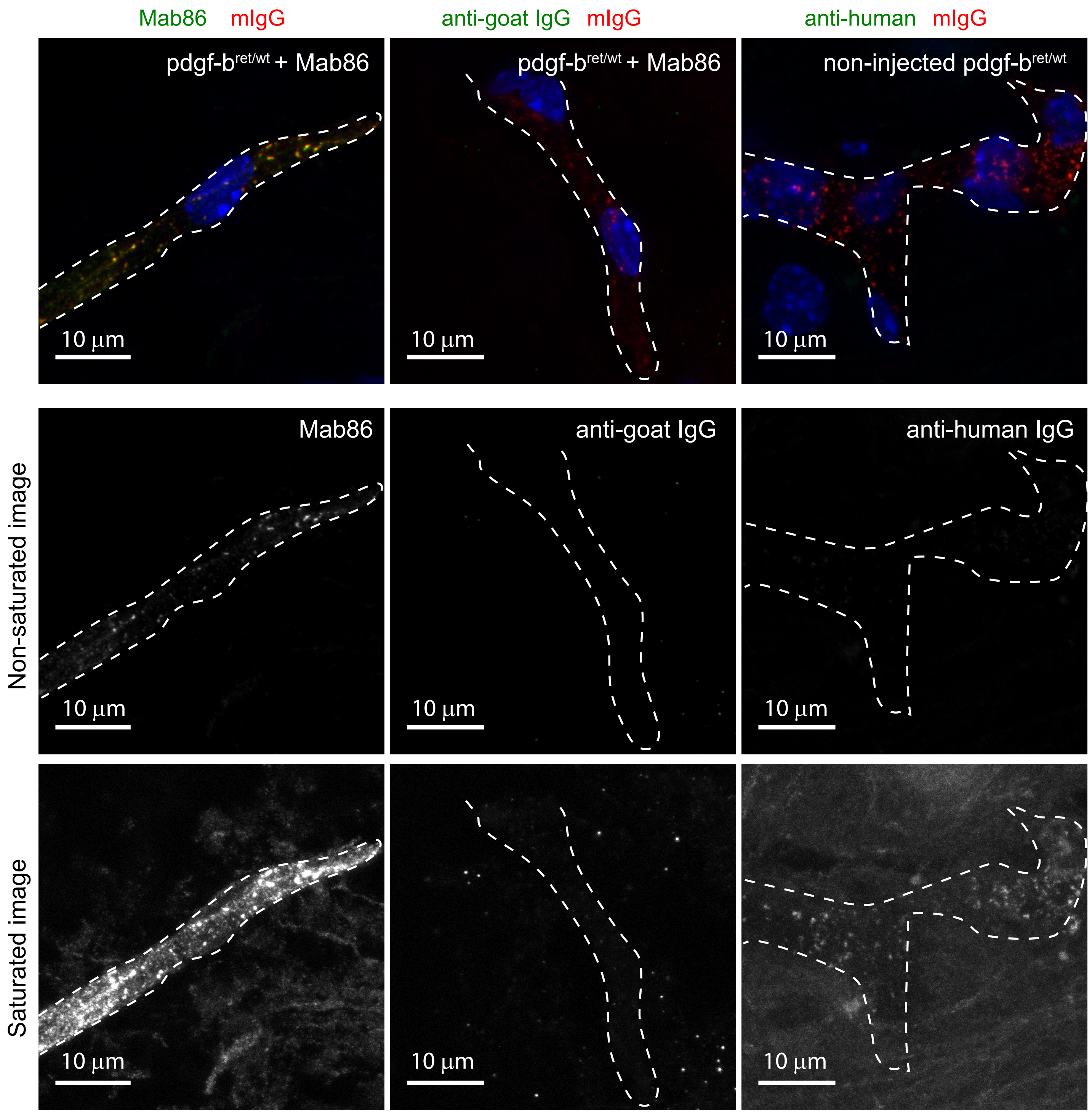

Supplementary Figure 5. Specific detection of Mab86 by anti-human IgG antibodies after acute injection. Representative high-resolution images of a cortical mouse microvessels showing that Mab86 vesicular structures (green) are only detected after immunostaining with a donkey anti-human IgG antibody (left panels) but not with a donkey anti-goat IgG antibody (central panel). Immunostaining with anti-human IgG antibody of non-injected animals revealed only background staining (right panel). Capillaries are identified by the mIgG signal in red. mIgG is shown in red and DAPI-stained nuclei in blue. The middle panel shows non-saturated greyscale images corresponding to Mab86, anti-goatIgG or anti-human IgG. The bottom panel shows saturated images.
